# Compassion fatigue: a challenge to comprehensive health care and SDG 3 under the 2030 Agenda

**DOI:** 10.1590/0034-7167-2025-0175

**Published:** 2026-03-16

**Authors:** Matheus Mendes Pereira, Mayara Karoline Silva Lacerda, Daniella Fagundes Souto, Joanilva Ribeiro Soares, Carla Silvana de Oliveira e Silva

**Affiliations:** I Universidade Estadual de Montes Claros. Montes Claros, Minas Gerais, Brazil

**Keywords:** Compassion Fatigue, Sustainable Development, Sustainable Development Goals, Comprehensive Health Care, Health Promotion., Desgaste por Empatía, Desarrollo Sostenible, Objetivos de Desarrollo Sostenible, Atención Integral de Salud, Promoción de la Salud.

## Abstract

**Objectives:**

to analyze the relationship between compassion fatigue among health care professionals and the implementation of Sustainable Development Goal 3 (SDG 3) under the 2030 Agenda for Sustainable Development, assessing this condition’s impact on the quality of care and the effectiveness of health services.

**Methods:**

scoping review performed according to JBI methodological guidance and the PRISMA-ScR checklist, with searches in PubMed, Scopus, Web of Science, and SciELO. Of the 245 studies identified, 20 were included in the final analysis.

**Results:**

findings suggest that compassion fatigue may compromise the quality of care, increase clinical errors and staff turnover, and reduce decision-making capacity. The absence of institutional support can intensify these impacts.

**Conclusions:**

institutional strategies are necessary to mitigate the effects of compassion fatigue among health care professionals and ensure safe, equitable care that aligns with the principles of the 2030 Agenda.

## RESUMEN

**Objetivos:**

analizar la relación entre la fatiga por compasión en profesionales de la salud y la implementación del ODS 3 (Agenda 2030), evaluando sus impactos en la calidad de la atención y la efectividad de los servicios de salud.

**Métodos:**

revisión de alcance según directrices del JBI Brasil y PRISMA-ScR, con búsquedas en PubMed, Scopus, *Web of Science* y SciELO. De 245 estudios identificados, 20 se incluyeron en el análisis final.

**Resultados:**

los hallazgos indican que la fatiga por compasión puede comprometer la calidad de la atención, aumentar errores clínicos y la rotación del personal, y reducir la capacidad de toma de decisiones. La ausencia de apoyo institucional puede intensificar estos efectos.

**Conclusiones:**

se requieren estrategias institucionales para mitigar la fatiga por compasión en profesionales de la salud y garantizar atención segura y equitativa, alineada con los principios de la Agenda 2030.

## INTRODUCTION

Compassion fatigue is a form of emotional and psychological exhaustion that develops in professionals who are continuously exposed to others’ suffering, reducing their capacity to provide empathetic and effective care^(1)^. Figley describes this phenomenon as a consequence of prolonged involvement with individuals in situations of vulnerability: “secondary traumatic stress” is a sudden, intense reaction arising from empathic exposure to the suffering of traumatized people, whereas burnout results from chronic workplace stressors such as overload and lack of support. Compassion fatigue emerges from the combination of these two phenomena, representing a significant risk to the mental health of professionals who care for people in distress^(1)^.

The impact of compassion fatigue extends beyond professionals’ well-being, affecting the quality of services delivered and, consequently, the effectiveness of public policies aimed at promoting health^(2)^. Cocker and Joss^(2)^ highlight that high levels of emotional exhaustion among frontline workers are directly associated with increased staff turnover, absenteeism, and deterioration in the care provided to patients. This problem becomes even more salient in the context of the United Nations 2030 Agenda, which, through Sustainable Development Goal 3 (SDG 3), commits to ensuring universal access to health care and promoting well-being at all ages^(3)^. However, when health care professionals face precarious conditions and excessive demand, achieving this goal becomes infeasible, as emotional exhaustion can disrupt continuity and lower the quality of health services^(2)^.

The relationship between compassion fatigue and SDG 3 becomes even clearer when examining the consequences of this phenomenon for patient safety. According to Duarte and Pinto-Gouveia^(4)^, professionals with advanced signs of emotional exhaustion are more prone to medical errors, communication difficulties with patients, and a marked reduction in decision-making capacity. These factors affect patients’ experience within the health system, increase the risk of adverse events, and call into question the effectiveness of care policies^(4)^. It is, therefore, essential to recognize compassion fatigue as a structural problem rather than merely an individual issue, avoiding solutions that exclusively shift responsibility to professionals while neglecting the working conditions that perpetuate this strain.

Mitigating this phenomenon requires measures that go beyond self-care strategies and promote structural changes in the work environment. For Figley^(1)^, implementing psychological support programs, reducing excessive workloads, and providing supportive spaces within institutions are fundamental steps to preserving professionals’ mental health. In addition, Duarte and Pinto-Gouveia^(4)^ stress the importance of institutional practices that value workers, coupled with continuing education programs that raise awareness about the profession’s psychosocial risks.

In this context, compassion fatigue should be understood not only as a phenomenon inherent to work in settings with high emotional demand, but also as a concrete barrier to achieving the global health targets set out in the 2030 Agenda. When health systems fail to provide adequate support for their professionals, the quality of care can be compromised, as can the sustainability of the service delivery model itself^(1-4)^. Including this issue in public policy guidelines aimed at promoting well-being is, therefore, indispensable, ensuring that universal health coverage is not an unattainable ideal but a feasible goal supported by healthy professionals who are emotionally prepared to perform their roles.

## OBJECTIVES

To analyze the relationship between compassion fatigue among health care professionals and the implementation of Sustainable Development Goal 3 (SDG 3) under the 2030 Agenda for Sustainable Development, assessing this condition’s impact on the quality of care and the effectiveness of health services.

## METHODS

### Ethical aspects

This study used publicly available data; therefore, ethics committee approval was not required.

### Study design

This is a scoping review conducted in accordance with recommendations from the Brazilian Center for Evidence-Based Health Care: a JBI Center of Excellence (JBI Brazil)^(5)^ and the Preferred Reporting Items for Systematic Reviews and Meta-Analyses Extension for Scoping Reviews (PRISMA-ScR) checklist^(6)^. This review aims to map key concepts in a defined area of research, identify knowledge gaps, and highlight the need for further studies. Based on JBI Brazil guidance, the main justifications for conducting this review include identifying the types of evidence available in the field under investigation, critically analyzing gaps in the scientific literature, and characterizing how factors associated with compassion fatigue affect the achievement of SDG 3 targets, which aim to promote health and well-being for all.

### Study planning

For this study’s planning, we used the stages proposed by Arksey and O’Malley^(7)^ and refined by Peters et al.^(8)^:

1. Define and align the research question.
2. Develop and align the inclusion criteria with the review objective.
3. Describe the planned approach to evidence searching, selection, data extraction, and presentation of results.
4. Search for the evidence.
5. Select the evidence.
6. Extract the data.
7. Analyze and synthesize the results.

### Search strategy

The search was structured using the PCC strategy (Population, Concept, and Context), recommended by JBI Brazil^(5)^. The population comprised health care professionals working in settings with high emotional demand. The concept of interest was compassion fatigue and the factors associated with this condition’s impact on the quality of care. The context involved implementing SDG 3 under the 2030 Agenda, which seeks to ensure health and well-being for all. The research question was: “How does compassion fatigue among health care professionals affect the quality of care and the effectiveness of health services? What are this phenomenon’s implications for implementing the targets of Sustainable Development Goal 3 (SDG 3) under the 2030 Agenda?”.

### Study period and setting

Data collection took place from January 20 to February 8, 2025, remotely, using personal electronic devices and without formal ties to academic institutions or institutional support. Access to databases occurred directly through publicly available platforms. We recognize that not using university libraries or academic platforms with restricted access may have limited the inclusion of studies available only via institutional subscription. Nevertheless, we sought to mitigate this limitation by rigorously applying methodological eligibility criteria, using structured search strategies and internationally recognized, open-access sources to ensure transparency and reproducibility in the study selection process.

Descriptors were retrieved from Health Sciences Descriptors (DeCS) and Medical Subject Headings (MeSH) to align searches in Portuguese and English. The terms were: “*Fadiga por compaixão*,” “*Desenvolvimento Sustentável*,” “*Objetivos do Desenvolvimento Sustentável* (ODS),” “*Assistência Integral à Saúde*,” “*Promoção da Saúde*,” “Compassion Fatigue,” “Sustainable Development,” “Sustainable Development Goals,” “Comprehensive Health Care,” and “Health Promotion.” For combining the descriptors, the Boolean operators “AND” and “OR” were used.

### Data sources

The following databases were consulted: PubMed, Scopus, Web of Science, and SciELO. These sources were selected for their scientific relevance, thematic coverage, and the methodological quality of the indexed studies.

The search strategy was adapted to each source’s specific features; however, descriptor combinations were preserved, and no time or language filters were applied (Chart 1).

**Chart 1:** Search syntax used across data sources, 2025

Database	Search strategy (Boolean operators)
**PubMed**	(“Compassion Fatigue”) AND (“Sustainable Development” OR “Sustainable Development Goals” OR “Comprehensive Health Care” OR “Health Promotion”)
**Scopus**	(“compassion fatigue”) AND (“sustainable development” OR “sustainable development goals” OR “comprehensive health care” OR “health promotion”)
**Web of Science**	(“compassion fatigue”) AND (“sustainable development” OR “sustainable development goals” OR “comprehensive health care” OR “health promotion”)
**SciELO**	(“*fadiga por compaixão*”) AND (“*desenvolvimento sustentável*” OR “*objetivos do desenvolvimento sustentável*” OR “*assistência integral à saúde*” OR “*promoção da saúde*”)

### Inclusion and exclusion criteria

We included full-text scientific articles, theses, dissertations, and guidelines that addressed the research question. Eligible studies examined the relationship between compassion fatigue among health care professionals and the implementation of Sustainable Development Goal 3 (SDG 3) under the 2030 Agenda-committed to ensuring health and well-being for all-assessing this condition’s impact on the quality of care and the effectiveness of health services. We excluded opinion pieces, letters to the editor, abstracts, and studies that did not align with the central topic of interest. Duplicate records or studies without full-text availability were also excluded.

### Study protocol

Two independent reviewers conducted the search, screening, and study inclusion. Any disagreements were resolved through an additional review or consultation with a third reviewer. The PAGER methodology^(7)^ was used to enhance methodological rigor and the clarity of result presentation, providing a consistent analytic approach that highlights Patterns, Advances, Gaps, Evidence for practice, and Research recommendations. Given this framework’s relevance, Table 1 was structured according to this model^(7)^.

### Analysis of results

Data from the included studies were organized in a Microsoft Excel spreadsheet. The analysis was descriptive and presented in charts to facilitate the interpretation of findings.

## RESULTS

The search strategies combined descriptors with Boolean operators to ensure broad yet precise retrieval of the literature. In total, 245 records were identified: 120 in PubMed, 80 in Scopus, 30 in Web of Science, and 15 in SciELO. After removing 125 duplicates, 120 unique records remained and were then screened.

In the initial screening, titles and abstracts of the 120 records were reviewed against predefined inclusion criteria. Exclusion criteria encompassed articles that did not address compassion fatigue in the context of health care delivery, studies examining occupational fatigue in professional groups unrelated to clinical practice, articles lacking a clear methodology or full-text availability, and publications that were not peer-reviewed. Records without sufficient methodological information to ensure reproducibility and reliability were also excluded. At this stage, 75 records were excluded.

The remaining 45 records then underwent a second screening based on an in-depth appraisal of titles and abstracts, which led to the exclusion of 25 records for failing to fully meet the review objectives-either due to the methodological approach taken or to the absence of information relevant to the proposed analysis.

Twenty records advanced to full-text assessment to determine eligibility based on methodological consistency and adherence to the study objectives.

At the end of the selection process, 20 sources were included: 17 peer-reviewed journal articles addressing compassion fatigue in the context of health care delivery, 2 specialized books that expanded the discussion of its impact on professional practice, and 1 institutional document.

**Figure 1:**
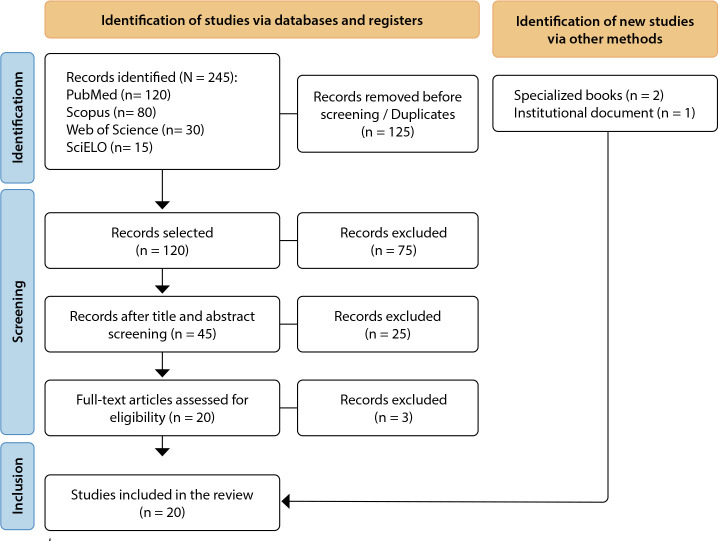
Flowchart of the search and study selection process

This corpus enabled an in-depth analysis of the impact of compassion fatigue on the quality of care and patient safety, while highlighting structural challenges that influence the implementation of SDG 3. The methodological rigor employed in searching and selecting sources enhances the reliability of the results, facilitating a comprehensive understanding of the factors associated with compassion fatigue among health care professionals and their implications for the health system. Figure 1 summarizes the search process.

Chart 2 synthesizes key factors that may reduce compassion fatigue, highlighting strategies already proposed for mitigation, gaps that remain underexplored in the literature, and implications for improving health care delivery.

**Chart 2:** PAGER^*^ structure derived from the analysis of the selected articles

Patterns (Associated factors)	Advances	Gaps	Evidence for practice	Research recommendations
Excessive workload	Studies have shown that reducing workload can help mitigate compassion fatigue and improve the quality of care.	Limited research on effective scheduling models that reconcile professional well-being with continuity of care.	Flexible shifts and regular breaks, with monitoring for exhaustion risk and clinician performance.	Investigate how different work-schedule formats affect mental health and professional performance.
Lack of institutional support	Some institutions have implemented psychological support programs, though often as isolated initiatives.	Low institutional uptake of debriefing and staff-support strategies, as well as a lack of standardized guidelines for emotional support.	Ongoing psychological support and supervision programs to strengthen retention and relational support.	Examine the impact of institutional support on reducing absenteeism and improving job satisfaction among health workers.
High staff turnover	Identification of strategies to improve staff retention and team stability.	A shortage of longitudinal studies analyzing the impact of turnover on the quality of care.	Teams showed a lower incidence of adverse events and greater treatment adherence.	Study how institutional practices can improve staff retention and patient safety.
Prolonged exposure to suffering	Emotional intelligence training has begun to be adopted in some settings to minimize emotional impacts.	A lack of public policies focused on protecting the emotional health of healthcare workers.	Resilience and emotion-regulation strategies, plus training aimed at reducing the risk of compassion fatigue.	Evaluate the effectiveness of emotion-focused training programs in reducing compassion fatigue.
COVID-19 pandemic impacts	Emergency measures were implemented to support professionals, albeit with limited reach.	Few studies assess the long-term mental health effects of the pandemic on professionals.	Institutional actions that provide ongoing support and scheduled recovery time are essential to reduce psychological harm.	Explore institutional strategies to mitigate the psychological impacts of the pandemic on frontline professionals.

* *PAGER - Patterns, Advances, Gaps, Evidence for practice, Research recommendations.*

The relationship between compassion fatigue and challenges to achieving SDG 3 targets (ensuring universal access to health and promoting well-being at all ages) becomes evident when examining the emotional and professional strain affecting healthcare workers. Chart 3 presents a synthesis of these elements, showing how structural and organizational factors influence professionals’ physical and mental health, as well as compromise the quality of care.

**Chart 3:** Factors associated with compassion fatigue and impact on SDG 3^*^

Associated factors	Impact on SDG 3
Excessive workload	Undermines professionals’ physical and mental health, lowering the quality of care and increasing the risk of preventable clinical errors.
Prolonged exposure to suffering	Reduces empathy and the humanization of care, hindering progress toward a universal, high-quality health system.
Lack of institutional support	Exacerbates the effects of compassion fatigue, contributing to workforce shortages and complicating the implementation of sustainable health systems.
High emotional demand	Increases vulnerability to psychological strain, disrupting continuity of care and patients’ adherence to treatment.
High staff turnover	Leads to fragmented care and unstable teams; consequently, implementing appropriate public health policies becomes more difficult.
Frequent contact with death	Reduces clinical decision-making capacity and leads to patient-safety failures, underscoring the need for adequate psychological support.
Lack of psychological support programs	Increases the incidence of mental disorders and work absences, reducing the quality of care and hindering the implementation of effective health policies.
Communication difficulties across teams	Impairs the execution of clinical protocols and compromises patient safety, thereby undermining the effectiveness of therapeutic interventions.
Mechanization of care delivery	Erodes the humanization of care: professionals withdraw from the therapeutic relationship, which harms patients’ adherence to treatment.
Sense of professional failure	Generates demotivation and decreased productivity, hindering the implementation of effective and sustainable health policies.
Lack of breaks and adequate rest periods	Impairs concentration and increases the risk of clinical errors, compromising patient safety.
Impacts of the COVID-19 pandemic	Intensifies compassion fatigue due to heavier workloads and collective bereavement, revealing gaps in institutional support for healthcare workers.

* *SDG - Sustainable Development Goal.*

Finally, Chart 4 synthesizes the included sources by reference, population, method, and outcomes.

**Chart 4:** Summary of the studies included in the scoping review

Year/Reference	Population	Method	Outcome
Figley^(1)^	Professionals who care for people in distress	Book	Defines and differentiates compassion fatigue; lists symptoms and risk factors; and proposes coping and prevention strategies for professionals exposed to others’ suffering.
Cocker F, Joss N (2016)^(2)^	Health care professionals in emergency and community services	Systematic review	Shows that compassion fatigue is common in these groups due to constant exposure to suffering and emotional overload; highlights the importance of institutional psychological support and workload reduction.
ONU (2015)^(3)^	Global scope	Official/institutional document	Presents the 17 Sustainable Development Goals (SDGs) of the 2030 Agenda.
Duarte J, Pinto-Gouveia J (2017)^(4)^	Nurses	Cross-sectional study	Excessive empathy and guilt are strongly associated with compassion fatigue and burnout; emotional self-regulation and professional training emerge as protective factors.
Garnett A, et al. (2023)^(9)^	Health care professionals	Scoping review	Identifies coping strategies for compassion fatigue and notes gaps in institutional actions, reinforcing the need for standardized guidelines.
Jilou V, et al. (2021)^(10)^	Health care professionals	Scoping review	Organizational strategies-such as emotional-intelligence training and psychological support-attenuate compassion fatigue and sustain the quality of care.
Cardoso AA, Tomotani DYV, Mucci S (2023)^(11)^	Professionals caring for patients at end of life	Qualitative study	Continuous exposure to suffering and death leads to progressive emotional exhaustion; the study suggests that peer support and clinical supervision are essential to preserve professionals’ mental health.
Chen, et al. (2022)^(12)^	Primary health care professionals	Cross-sectional study	It is essential to strengthen institutional support and psychological resources to reduce burnout and prevent professionals from leaving the workforce.
Phillips CS, Volker DL (2020)^(13)^	Oncology nurses	Qualitative study	Emotional overload and prolonged exposure to suffering cause significant mental strain; the study stresses institutional practices that balance emotional engagement with healthy professional detachment.
Mason VM, et al. (2014)^(14)^	Trauma and surgical ICU nurses	Pilot study	Compassion fatigue is linked to suffering and work overload; a psychological support program can strengthen emotional resilience.
Zambrano SC, et al. (2014)^(15)^	Palliative-medicine specialists	Qualitative study	Continuous exposure to death adversely affects professionals’ health, resulting in high exhaustion and reduced empathy; periodic psychological supervision is recommended.
Pereira SM, et al. (2016)^(16)^	Intensive care professionals	National comparative study	Working in intensive care doubles the odds of exhaustion and compassion fatigue compared with palliative care; interventions are needed to reduce emotional burden.
Chang WP (2018)^(17)^	Clinical nurses	Quantitative study	Social support improves nurses’ capacity to cope with patient death and compassion fatigue; its absence is associated with increased staff turnover.
Oliver A, et al. (2021)^(18)^	Palliative care professionals	Cross-cultural study	Self-care is crucial for mitigating the effects of compassion fatigue and enhancing quality of life; institutions should promote actions that promote workers’ well-being.
Ko W, Kiser-Larson N (2016)^(19)^	Oncology nurses	Quantitative study	Prolonged exposure to severe cancer cases leads to high occupational stress and fatigue; institutional psychological support policies are fundamental for oncology staff.
Laor-Maayany R, et al. (2020)^(20)^	Oncologists	Cross-sectional study	Poorly processed grief and a sense of failure when caring for terminal patients aggravate compassion fatigue; clinical supervision and support groups may minimize this impact.
Pattison N, et al. (2020)^(21)^	Health care professionals in critical and palliative care	Qualitative study	Burnout and emotional exhaustion directly impair the delivery of humane, patient-centered care; institutional strategies are necessary to preserve workers’ mental health.
Moreira RS (2021)^(22)^	Health care professionals in the context of COVID-19	Quantitative study	The pandemic increased levels of compassion fatigue due to workload and deaths; psychological support protocols may reduce long-term emotional harm.
Lago^(23)^	Health care professionals caring for people with physical and psychological suffering	Qualitative study; literature review	Analyzes mental suffering among health care professionals, deepens the concept of compassion fatigue, and proposes both institutional and individual coping strategies.
Lago^(24)^	Health care professionals in pediatric palliative care	Narrative literature review	Discusses ethical, cultural, clinical, and organizational challenges in pediatric palliative care in Brazil and worldwide.

## DISCUSSION

### Compassion fatigue as a barrier to the quality of care and to SDG 3

Compassion fatigue, defined as exhaustion resulting from continuous exposure to others’ suffering, is a key driver of deteriorating mental health among health care professionals, affecting their individual well-being, the quality of services delivered, and patient safety^(1)^. Over the past decade, studies have shown that its impacts extend beyond the subjective sphere, as evidenced by increased absenteeism, heightened turnover, and the deterioration of interpersonal relationships within organizations^(2)^. In a context of structural overload and scarce institutional support, compassion fatigue not only reduces professionals’ capacity to respond effectively to patients’ needs but also increases the number of adverse events in care^(3,4,9)^.

The prevalence of compassion fatigue among health care professionals is associated with multiple organizational factors, including long shifts, excessive caseloads, and repeated exposure to emotionally challenging situations^(10)^. In the pandemic context, this problem has become even more concerning, as system strain and intensified service demands exacerbated exhaustion and psychological distress among professionals^(11)^. Garnett et al.^(9)^ report that frontline workers in the COVID-19 response showed increased levels of compassion fatigue due to prolonged exposure to human suffering without adequate institutional support.

Beyond individual repercussions, evidence links compassion fatigue to a higher propensity for medical errors, a decline in the quality of care, and intensified feelings of depersonalization and cynicism among health workers^(12,23)^. SDG 3 underscores the importance of ensuring patient safety and reducing mortality from preventable causes; however, when professionals experience high levels of emotional exhaustion, their capacity to deliver effective, humanized care is drastically reduced^(13)^. Worker exhaustion, therefore, cannot be dissociated from global health targets, since its impact on the quality of care diminishes the effectiveness of public policies aimed at population well-being^(14)^.

Health professionals’ vulnerability to compassion fatigue is not restricted to hospital settings. According to Cardoso, Tomotani, and Mucci^(11)^, those working in palliative care and intensive care units exhibit high levels of psychological distress, intensified by repeated contact with death and the need to make complex decisions under intense pressure. The absence of institutional strategies to mitigate this phenomenon contributes to a cycle of progressive strain in which emotional exhaustion becomes a determining factor for work leave and early exit from the profession^(15)^. The loss of qualified professionals undermines continuity of care and exacerbates inequities in access to health services, hindering the implementation of SDG 3 targets and weakening the sustainability of service delivery systems.

The relationship between compassion fatigue and achieving Sustainable Development Goal 3 (SDG 3) is evident because team stability is a pillar of equitable, high-quality care^(16,17)^. The 2030 Agenda, proposed by the United Nations (UN), states that access to health must go beyond expanding services to include safe, humanized, and sustainable care^(3)^. Compassion fatigue undermines this aim: exhausted professionals tend to exhibit reduced empathy, impaired clinical communication, and a greater propensity for care-related errors; these problems are aggravated in high-demand settings such as emergency departments and intensive care units, where emotional overload reaches extreme levels and opportunities for psychological recovery are scarce^(10,11,18,24)^.

The impacts of compassion fatigue on patient safety are well-documented. Ko and Kiser-Larson^(19)^ found that institutions with higher levels of this phenomenon among professionals face rising absenteeism, turnover, and fragmented care, which impairs continuity of care. Team turnover, for its part, directly influences patients’ adherence to treatment, since a weakened therapeutic bond reduces trust in the health system and hampers the implementation of long-term therapeutic strategies^(20)^.

Studies conducted between 2020 and 2021 revealed that health care professionals with high levels of compassion fatigue also reported lower job satisfaction and diminished professional commitment, along with a greater intention to leave their organizations, as reflected in higher turnover rates. In another context, compassion fatigue negatively affected nurses’ behavioral intentions when caring for patients with COVID-19, which can undermine the quality of care delivered^(9)^.

When health care professionals do not receive adequate support, the risk of burnout increases and care tends to become mechanized, moving practice away from the principles of SDG 3, which emphasize patient-centered care and population well-being^(12)^.

Emotional overload compromises both the quality of care and the safety of clinical processes, creating an environment that is more prone to errors. Mason et al.^(14)^ report that professionals subjected to prolonged emotional exhaustion show reduced decision-making capacity, poorer clinical judgment, and greater vulnerability to errors in medication administration, test interpretation, and device management. Emotional overload weakens professionals’ cognition, making them less attentive to subtle signs that could indicate patient deterioration^(16)^.

According to Garnett et al.^(9)^, the negative effects of the COVID-19 pandemic on the mental health and well-being of health care professionals continue to limit their activities and functions, contributing to the development of compassion fatigue. Shortages of personal protective equipment intensified the spread of the virus and heightened the fear of contracting and transmitting the disease to colleagues, patients, and family members. This scenario led to more absences, prolonged sick leave, and higher staff turnover, while overload intensified amid staffing shortages, constant workflow changes, and the continuous care of critically ill patients, often with fatal outcomes.

Thus, institutional neglect of workers’ well-being not only affects occupational health but also undermines the effectiveness of therapeutic interventions and patient safety.

Implementing institutional strategies to reduce compassion fatigue is fundamental to achieving SDG 3. Garnett et al.^(9)^ emphasize that policies to reduce workload, expand psychological support, and train professionals in emotional coping strategies can yield significant improvements in workforce retention and the quality of care. Cardoso, Tomotani, and Mucci^(11)^ note that compassion fatigue is directly linked to increased mortality from preventable causes, as exhausted professionals exhibit a reduced capacity to respond to emergencies and show less assertiveness in clinical decision-making. Ensuring that health care workers practice under adequate conditions is, therefore, not merely an occupational matter but an essential strategy for reducing morbidity and mortality and fulfilling the principles set forth under SDG 3^(23)^.

In addition, compassion fatigue undermines equity in access to health services by weakening systems’ ability to maintain stable, sufficiently trained teams to meet the growing needs of the population. Chen et al.^(12)^ analyzed how emotional exhaustion affects primary care professionals: where workload is high and institutional support is insufficient, staff turnover leads to discontinuity of care and unequal access to essential services. This situation is especially concerning in lowand middle-income countries, where shortages of health professionals already challenge efforts to achieve universal access to care^(17)^.

The implementation of public and organizational policies to prevent compassion fatigue should be treated as a central pillar for realizing SDG 3. Figley^(1)^ argues that professionals’ emotional resilience should not be framed as an individual responsibility, but rather as a collective endeavor that entails institutional change and stronger practices to protect mental health in the workplace. Including psychological support programs, training in the management of occupational stress, and measures that promote more balanced work schedules is essential to ensure that workers are physically and emotionally prepared to perform their roles ethically, safely, and effectively^(21)^.

Transforming this scenario requires the commitment of managers, policymakers, and health education institutions to promote a care model that ensures the principles of SDG 3 apply not only to the populations served but also to the professionals responsible for delivering that care. Beyond expanding access, the sustainability of service delivery systems depends on strategies that build a work environment that protects professionals from exhaustion and enables the continuity of safe, humanized care aligned with society’s needs^(21,22)^.

Therefore, addressing compassion fatigue extends beyond attending to health workers’ needs: it is essential for the sustainability of service delivery systems and for building a model of care consistent with the principles of SDG 3. Only structured, sustainable strategies will ensure that the global commitment to health and well-being for all moves from aspiration to an equitable, attainable reality for all populations.

### Study limitations

This study has several limitations. First, not all factors related to compassion fatigue and its intersection with Sustainable Development Goal 3 (SDG 3) were addressed specifically and thoroughly in the reviewed literature. We also observed a scarcity of clinical trials and systematic reviews directly focused on the topic, which limited the identification of solid, conclusive evidence on effective strategies to mitigate compassion fatigue. This limitation was compounded by restricted access to the full text of some articles retrieved from the databases. In addition, heterogeneity across the included studies may have affected the consistency and applicability of the findings, especially given the distinct characteristics of the health systems represented.

### Contributions to the field

This study provides a detailed overview of factors associated with compassion fatigue and expands understanding of how this phenomenon directly affects the implementation of SDG 3, particularly in relation to the quality of care and patient safety. It contributes by identifying knowledge gaps that can guide future research and by highlighting the need for structural, workplace-level strategies to support health care professionals. It also underscores the importance of incorporating compassion fatigue into public health policy, emphasizing improved working conditions and the promotion of workers’ mental health to ensure the effectiveness of global health and well-being targets.

## FINAL CONSIDERATIONS

This study demonstrates that compassion fatigue poses a significant challenge to the sustainability and quality of health systems, directly affecting health care professionals, patients, and the achievement of Sustainable Development Goal 3 (SDG 3). Emotional and psychological strain among workers compromises patient safety, increases preventable clinical errors, and contributes to fragmented care, showing that the issue goes beyond the individual level and reflects structural shortcomings in work environments.

The findings suggest that compassion fatigue is linked to factors such as long shifts, inadequate institutional support, and prolonged exposure to others’ suffering; these conditions are associated with increased staff turnover and absenteeism, as well as declines in the quality of care. In overburdened health systems, as observed during the COVID-19 pandemic, these issues become even more concerning.

The intersection between compassion fatigue and SDG 3 underscores the need for structural strategies to mitigate its impacts. Achieving global health targets requires not only expanding access to services but also ensuring conditions that enable professionals to perform their roles safely and effectively. The absence of initiatives to protect workers’ mental health can jeopardize service sustainability and the quality of care provided.

Accordingly, this study reinforces the importance of institutional policies to prevent compassion fatigue. Measures such as reducing workload, providing psychological support, training in emotional coping strategies, and fostering a more supportive, humane work environment are essential to minimize its impact. Implementing these actions can enhance the quality of care, reduce professionals’ vulnerability, and promote a more resilient and equitable health system.

Addressing compassion fatigue is not only a matter of occupational well-being; it is also a prerequisite for effective public health policy. Only the collective commitment of managers, policymakers, and health care professionals will ensure that SDG 3-grounded in the commitment to universal access to health care and the promotion of well-being for all-becomes not merely a global ideal, but a reality accessible to all populations.

## Data Availability

The research data are available within the article.
