# Erratum: Compassion fatigue: a challenge to comprehensive health care and SDG 3 under the 2030 Agenda

**DOI:** 10.1590/0034-7167.202679e0320250175

**Published:** 2026-08-07

In the article “Compassion fatigue: a challenge to comprehensive health care and SDG 3 under the 2030 Agenda”, with DOI number: https://doi.org/10.1590/0034-7167-2025-0175, published in Revista Brasileira de Enfermagem, 2026;79:e20250175, on page 8:

Where it read:

**FUNDING**

Fundação de Amparo à Pesquisa do Estado de Minas Gerais (FAPEMIG).

Lê-se:

**FUNDING**

Research Support Foundation of the State of Minas Gerais (FAPEMIG). Process APQ-04716-24, approved by Public Notice No. 009/2024 - Strengthening and consolidation of research at UEMG and UNIMONTES.

Prof. PhD Dulce Barbosa

*Editor in Chief*

