# Supplementary material for: Compassion fatigue: a challenge to comprehensive health care and SDG 3 under the 2030 Agenda
Source: Rev Bras Enferm. 2026 Mar 16;79:e20250175. doi: 10.1590/0034-7167-2025-0175 (PMC12995330; doi:10.1590/0034-7167-2025-0175)
Supplement: Translation

# Translation: Fadiga por compaixão: desafio à saúde integral e ao ODS 3 da Agenda 2030

**DOI:** 10.1590/0034-7167-2025-0175pt

**Authors:** Matheus Mendes Pereira, Mayara Karoline Silva Lacerda, Daniella Fagundes Souto, Joanilva Ribeiro Soares, Carla Silvana de Oliveira e Silva

**Affiliations:** I Universidade Estadual de Montes Claros. Montes Claros, Minas Gerais, Brasil

**Keywords:** Fadiga por Compaixão, Desenvolvimento Sustentável, Objetivos do Desenvolvimento Sustentável, Assistência Integral à, Saúde, Promoção da Saúde.

## Abstract

**Objetivos:**

analisar a relação entre a fadiga por compaixão em profissionais de saúde e a implementação do Objetivo de Desenvolvimento Sustentável 3 (ODS 3) da Agenda 2030, avaliando os impactos dessa condição na qualidade da atenção e na efetividade dos serviços de saúde.

**Métodos:**

revisão de escopo conforme diretrizes do JBI Brasil e checklist PRISMA-ScR, com buscas nas bases PubMed, Scopus, *Web of Science* e SciELO. Dos 245 estudos identificados, 20 foram incluídos na análise final.

**Resultados:**

os achados apontam que a fadiga por compaixão pode comprometer a qualidade do cuidado, aumentar erros clínicos e rotatividade de profissionais e reduzir a capacidade de decisão. A ausência de suporte institucional pode intensificar esses impactos.

**Conclusões:**

são necessárias estratégias institucionais para mitigar os efeitos da fadiga por compaixão nos profissionais de saúde e garantir uma atenção segura, equitativa e alinhada aos princípios da Agenda 2030.

## INTRODUÇÃO

A fadiga por compaixão constitui uma forma de exaustão emocional e psicológica que se desenvolve em profissionais expostos continuamente ao sofrimento alheio, diminuindo sua capacidade de oferecer um cuidado empático e eficaz^(1)^. Figley descreve esse fenômeno como uma consequência do envolvimento prolongado com indivíduos em situações de vulnerabilidade: o “estresse traumático secundário” é uma reação súbita e intensa decorrente da exposição empática ao sofrimento de pessoas traumatizadas, enquanto o *burnout* resulta de estressores crônicos no ambiente de trabalho, como sobrecarga e falta de suporte. A fadiga por compaixão emerge da combinação desses dois fenômenos, representando um risco significativo para a saúde mental dos profissionais que cuidam de pessoas em sofrimento^(1)^.

O impacto da fadiga por compaixão transcende o bem-estar dos profissionais, afetando a qualidade dos serviços prestados e, consequentemente, a efetividade das políticas públicas voltadas à promoção da saúde^(2)^. Cocker e Joss^(2)^ destacam que altos níveis de exaustão emocional entre trabalhadores da linha de frente estão diretamente relacionados ao aumento da rotatividade profissional, ao absenteísmo e à deterioração da assistência prestada aos pacientes. Esse problema torna-se ainda mais relevante no contexto da Agenda 2030 das Nações Unidas, que estabelece, por meio do Objetivo de Desenvolvimento Sustentável 3 (ODS 3), o compromisso de assegurar acesso universal à saúde e promover o bem-estar em todas as idades^(3)^. Entretanto, quando profissionais de saúde são submetidos a condições precárias e a uma demanda excessiva, torna-se inviável implementar esse objetivo, uma vez que o esgotamento emocional pode gerar quebra na continuidade e queda da qualidade dos serviços de saúde^(2)^.

A relação entre a fadiga por compaixão e o ODS 3 torna-se ainda mais evidente ao se analisarem as consequências desse fenômeno sobre a segurança do paciente. Segundo Duarte e Pinto-Gouveia^(4)^, profissionais que apresentam sinais avançados de exaustão emocional demonstram maior propensão a erros médicos, dificuldades na comunicação com os pacientes e uma redução significativa na capacidade de tomar decisões clínicas assertivas. Esses fatores atingem a experiência do paciente no sistema de saúde, o que aumenta o risco de eventos adversos e coloca em xeque a efetividade das políticas de cuidado^(4)^. Assim, torna-se essencial reconhecer a fadiga por compaixão como um problema estrutural e não só como uma questão individual, evitando soluções que transfiram a responsabilidade exclusivamente para o profissional e negligenciem as condições laborais que perpetuam esse desgaste.

Para minimizar os impactos desse fenômeno, é necessário adotar medidas que não se limitem a estratégias de autocuidado, mas que promovam mudanças estruturais no ambiente de trabalho. Para Figley^(1)^, a implementação de programas de suporte psicológico, a redução de jornadas excessivas e a oferta de espaços de acolhimento dentro das instituições são medidas fundamentais para preservar a saúde mental dos profissionais. Além disso, Duarte e Pinto-Gouveia^(4)^ ressaltam a importância de práticas institucionais que valorizem o trabalhador, aliadas a programas de educação continuada que promovam a conscientização sobre os riscos psicossociais da profissão.

Diante desse cenário, a fadiga por compaixão precisa ser compreendida não só como um fenômeno inerente ao trabalho em contextos de alta demanda emocional, mas também como um possível entrave real à efetivação das metas de saúde global estabelecidas na Agenda 2030. Quando sistemas de saúde falham em oferecer suporte adequado a seus profissionais, a qualidade da assistência prestada pode ser prejudicada, bem como a sustentabilidade do próprio modelo de atendimento^(1-4)^. Assim, incluir essa questão nas diretrizes de políticas públicas voltadas à promoção do bem-estar torna-se indispensável, garantindo que a universalização da saúde não seja um ideal inatingível, mas uma meta factível sustentada por profissionais saudáveis e emocionalmente preparados para o exercício de suas funções.

## OBJETIVOS

Analisar a relação entre a fadiga por compaixão em profissionais de saúde e a implementação do Objetivo de Desenvolvimento Sustentável 3 (ODS 3) da Agenda 2030 para o Desenvolvimento Sustentável, avaliando os impactos dessa condição na qualidade da atenção e na efetividade dos serviços de saúde.

## MÉTODOS

### Aspectos éticos

Este estudo foi realizado com base em dados de domínio público, portanto não foi necessário submetê-lo ao Comitê de Ética em Pesquisa.

### Desenho do estudo

Trata-se de uma revisão de escopo, conforme as recomendações do Centro Brasileiro para o Cuidado à Saúde Baseado em Evidências: Centro de Excelência do JBI (JBI Brasil)^(5)^ e o checklist *Preferred Reporting Items for Systematic Reviews and Meta-Analyses Extension for Scoping Review* (PRISMA-ScR)^(6)^. A presente revisão tem como finalidade mapear os principais conceitos relacionados a uma determinada área de pesquisa, identificar lacunas existentes no conhecimento e destacar a necessidade de novos estudos. Com base nas diretrizes do JBI Brasil, as principais justificativas para a elaboração desta revisão incluem: a identificação dos tipos de evidências disponíveis no campo investigado; a análise crítica das lacunas no conhecimento científico; e a caracterização sobre como os fatores associados à fadiga por compaixão impactam a efetivação das metas do ODS 3, que preconiza a promoção da saúde e o bem-estar para todos.

### Planejamento do estudo

Para o planejamento deste estudo, foram utilizadas as seguintes etapas propostas por Arksey e O’Malley^(7)^ e aprimoradas por Peters et al.^(8)^:

1. Definição e alinhamento da pergunta de pesquisa;
2. Desenvolvimento e alinhamento dos critérios de inclusão com o objetivo da pesquisa;
3. Definição da abordagem para busca das evidências, seleção dos estudos, extração de dados e apresentação dos resultados;
4. Realização da busca das evidências;
5. Seleção dos estudos;
6. Extração dos dados;
7. Análise e síntese dos resultados.

### Estratégia de pesquisa

A pesquisa foi formulada com base na estratégia PCC (População, Conceito e Contexto) recomendada pelo JBI Brasil^(5)^. A população incluída foi composta por profissionais da saúde expostos a situações de alta demanda emocional. O conceito de interesse foi a fadiga por compaixão e os fatores associados ao impacto dessa condição na qualidade dos serviços de saúde. O contexto envolveu a implementação do ODS 3 da Agenda 2030, que busca assegurar saúde e bem-estar para todos. A questão de pesquisa formulada foi: “Como a fadiga por compaixão em profissionais de saúde impacta a qualidade da atenção e a efetividade dos serviços de saúde? Quais são as implicações desse fenômeno para a implementação das metas do Objetivo de Desenvolvimento Sustentável 3 (ODS 3) da Agenda 2030?”.

### Período e local do estudo

A coleta de dados foi realizada entre 20 de janeiro e 08 de fevereiro de 2025, de forma remota, mediante dispositivos eletrônicos pessoais e sem vínculo formal com instituições acadêmicas ou apoio institucional. O acesso às bases de dados foi realizado diretamente por meio de plataformas de domínio público. Reconhece-se como limitação a não utilização de bibliotecas universitárias ou de plataformas acadêmicas com acesso restrito, o que pode ter restringido o alcance a determinados estudos disponíveis apenas mediante assinatura institucional. No entanto, a pesquisa buscou mitigar essa limitação por meio da aplicação rigorosa dos critérios metodológicos de elegibilidade, com uso de estratégias de busca estruturadas e fontes reconhecidas internacionalmente pela relevância e acesso aberto, assegurando a transparência e reprodutibilidade do processo de seleção dos estudos incluídos.

Os descritores foram consultados no Descritores em Ciências da Saúde (DeCS) e *Medical Subject Headings* (MeSH), objetivando adequar as buscas ao português e inglês. Foram estes: “Fadiga por compaixão”, “Desenvolvimento Sustentável”, “Objetivos do Desenvolvimento Sustentável (ODS)”, “Assistência Integral à Saúde”, “Promoção da Saúde”, “*Compassion Fatigue*”, “*Sustainable Development*”, “*Sustainable Development Goals*”, “*Comprehensive Health Care*” e “*Health Promotion*”. Para o cruzamento dos descritores, foram utilizados os operadores booleanos “AND” e “OR”.

### Fontes de dados

Foram consultadas as bases de dados PubMed, Scopus, Web of Science e SciELO. A seleção dessas fontes baseou-se em critérios de relevância científica, abrangência temática e qualidade metodológica dos estudos indexados.

A estratégia de busca foi adaptada de acordo com as especificidades de cada fonte utilizada, no entanto conservaram-se as combinações entre os descritores, e não foram adicionados filtros de restrição de tempo e idioma (Quadro 1).

**Quadro 1:** Sintaxe de busca dos artigos nas fontes de dados, 2025

Base de dados	Estratégia de busca (com operadores booleanos)
**PubMed**	(“*Compassion Fatigue*”) *AND* (“*Sustainable Development*” *OR* “*Sustainable Development Goals*” *OR* “*Comprehensive Health Care*” *OR* “*Health Promotion*”)
**Scopus**	(“*compassion fatigue*”) *AND* (“*sustainable development*” *OR* “*sustainable development goals*” *OR* “*comprehensive health care*” *OR* “*health promotion*”)
***Web of Science***	(“*compassion fatigue*”) *AND* (“*sustainable development*” *OR* “*sustainable development goals*” *OR* “*comprehensive health care*” *OR* “*health promotion*”)
**SciELO**	(“fadiga por compaixão”) *AND* (“desenvolvimento sustentável” *OR* “objetivos do desenvolvimento sustentável” *OR* “assistência integral à saúde” *OR* “promoção da saúde”)

### Critérios de inclusão e exclusão

Foram incluídos artigos científicos completos, dissertações, teses e diretrizes que respondessem à questão da pesquisa proposta. Os estudos selecionados deveriam abordar a relação entre a fadiga por compaixão em profissionais de saúde e a implementação do Objetivo de Desenvolvimento Sustentável 3 (ODS 3) da Agenda 2030, pautado no compromisso de assegurar acesso universal à saúde e promover o bem-estar em todas as idades, avaliando os impactos dessa condição na qualidade da atenção e na efetividade dos serviços de saúde. Foram excluídos artigos de opinião, cartas ao editor, resumos e estudos que não correspondessem ao tema central da pesquisa. Também foram excluídos registros duplicados ou artigos que não estivessem disponíveis integralmente.

### Protocolo do estudo

Dois avaliadores independentes realizaram a busca, triagem e inclusão dos estudos. As divergências foram resolvidas por uma revisão adicional ou por consulta a um terceiro avaliador. A metodologia PAGER^(7)^ foi utilizada para garantir maior rigor metodológico à presente revisão e maior clareza na apresentação dos resultados, por meio de uma abordagem analítica consistente que evidenciasse Padrões, Avanços, Lacunas, Evidências para a prática e Recomendações para pesquisa. Diante da relevância desse instrumento, o Quadro 2 foi estruturada com base nesse modelo^(7)^.

**Quadro 2:** Estrutura do PAGER^*^ obtida da análise dos artigos selecionados

Fator associado	Avanços	Lacunas	Evidências para prática	Recomendações de pesquisa
Carga horária excessiva	Estudos demonstram que a redução da carga de trabalho pode minimizar a fadiga por compaixão e melhorar a qualidade assistencial.	Pouca investigação sobre modelos eficazes de escalas que conciliam bem-estar profissional e continuidade da assistência.	Turnos flexíveis e pausas regulares, mantendo sob controle os índices de exaustão entre profissionais de saúde.	Avaliar como diferentes formatos de jornada de trabalho impactam a saúde mental e o desempenho dos profissionais.
Falta de suporte institucional	Algumas instituições implementaram programas de suporte psicológico, mas ainda de forma isolada.	Baixa adesão institucional a estratégias de acolhimento e deficiência de diretrizes padronizadas para suporte emocional.	Programas contínuos de apoio psicológico e supervisão para melhorar retenção profissional.	Explorar o impacto do suporte institucional na redução dos afastamentos e na satisfação dos trabalhadores da saúde.
Rotatividade elevada de profissionais	Identificação de estratégias para retenção de profissionais e melhoria da estabilidade das equipes.	Falta de estudos longitudinais que analisem o impacto da alta rotatividade na qualidade assistencial.	As equipes apresentaram menor incidência de eventos adversos e maior adesão ao tratamento.	Investigar como as políticas institucionais podem melhorar a retenção dos profissionais e a segurança do paciente.
Exposição prolongada ao sofrimento	Treinamentos em inteligência emocional começam a ser adotados em algumas instituições para minimizar impactos emocionais.	Falta de políticas públicas voltadas para a proteção emocional dos trabalhadores da saúde.	Estratégias de capacitação e resiliência emocional, bem como treinamento para a redução dos efeitos da fadiga por compaixão.	Avaliar a eficácia de programas de capacitação emocional na redução da fadiga por compaixão.
Impactos da pandemia de COVID-19	As medidas emergenciais foram empregadas para suporte aos profissionais, mas com abrangência limitada.	Poucos estudos avaliam os efeitos em longo prazo da pandemia na saúde mental dos profissionais.	A instituição oferecer suporte contínuo e pausas laborais para recuperação emocional é essencial para reduzir os danos psicológicos.	Explorar estratégias institucionais para minimizar os impactos psicológicos da pandemia em profissionais da linha de frente.

* *PAGER - Patterns, Advances, Gaps, Evidence for practice, Research recommendations.*

### Análise dos resultados

Os dados dos estudos selecionados foram organizados em uma planilha do Microsoft Excel. A análise foi conduzida de forma descritiva e apresentada em quadros para facilitar a compreensão dos achados.

## RESULTADOS

As estratégias de busca foram elaboradas utilizando a combinação dos descritores por meio de operadores booleanos para garantir a identificação ampla e precisa de estudos disponíveis. Como resultado, foram identificados 245 registros: 120 registros na PubMed, 80 na Scopus, 30 na *Web of Science* e 15 na SciELO. Após a remoção de 125 registros duplicados, restaram 120 estudos únicos, os quais seguiram para a etapa de rastreamento.

Na triagem inicial, os 120 registros foram submetidos à leitura dos títulos e resumos com o objetivo de verificar sua adequação aos critérios de inclusão previamente estabelecidos. Os critérios de exclusão englobaram artigos que não abordavam diretamente a fadiga por compaixão no contexto da assistência à saúde, estudos que analisavam a fadiga ocupacional em outros grupos profissionais sem relação com a prática clínica, artigos que não apresentavam metodologia clara ou que não disponibilizavam acesso ao texto completo, além de publicações que não haviam sido submetidos à revisão por pares. Foram excluídos também estudos que não forneciam informações metodológicas suficientes para garantir sua reprodutibilidade e confiabilidade na análise. Nessa etapa, foram excluídos 75 estudos.

Após a aplicação dos critérios, os 45 estudos remanescentes foram submetidos a uma nova triagem com base na leitura crítica e aprofundada dos títulos e resumos, resultando na exclusão de 25 registros por não atenderem integralmente aos objetivos da revisão, seja pela abordagem metodológica desenvolvida, seja pela ausência de informações relevantes para a análise proposta.

Com a exclusão de 5 estudos, os 20 restantes foram submetidos à leitura completa, avaliando sua elegibilidade com base na consistência do método e aderência aos objetivos do estudo.

Ao final do processo de seleção, 20 estudos foram incluídos na revisão de escopo: 17 publicados em periódicos indexados e revisados por pares abordando a fadiga por compaixão no contexto da assistência à saúde; 2 livros especializados que ampliavam a discussão sobre os impactos da fadiga por compaixão na prática profissional; e 1 documento institucional.

Esse corpus possibilitou uma análise aprofundada dos impactos da fadiga por compaixão sobre a qualidade assistencial e a segurança do paciente, além de evidenciar os desafios estruturais que influenciam a implementação das diretrizes do ODS 3. O rigor metodológico adotado na busca e seleção dos estudos assegura a confiabilidade dos resultados apresentados, permitindo uma compreensão abrangente dos fatores associados à fadiga por compaixão entre profissionais de saúde e suas implicações para o sistema. A Figura 1 sintetiza o processo de busca.

**Figura 1:** Fluxograma do processo de busca e seleção dos estudos

O Quadro 2 sintetiza os principais fatores que podem reduzir a fadiga por compaixão, destacando as estratégias já indicadas para sua mitigação, os aspectos ainda pouco explorados na literatura e as implicações para a melhoria da assistência em saúde.

A relação entre a fadiga por compaixão e os desafios à efetivação das metas do ODS 3 (Assegurar acesso universal à saúde e promover o bem-estar em todas as idades) torna-se evidente quando analisamos os fatores de desgaste emocional e profissional dos trabalhadores da saúde. O Quadro 3 apresenta uma síntese desses elementos, demonstrando como aspectos estruturais e organizacionais influenciam a saúde física e mental dos profissionais, além de comprometerem a qualidade da assistência prestada.

**Quadro 3:** Fatores associados à fadiga por compaixão e impacto no ODS 3^*^

Fator associado	Impacto no ODS 3
Carga horária excessiva	Debilita a saúde física e mental dos profissionais. Com isso, diminui a qualidade da assistência e aumenta o risco de erros clínicos evitáveis.
Exposição prolongada ao sofrimento	Diminui a empatia e a humanização do atendimento, dificultando a concretização de um sistema de saúde universal e de qualidade.
Falta de suporte institucional	Agrava os efeitos da fadiga por compaixão, o que contribui para a escassez de profissionais envolvidos e dificulta a implementação de sistemas de saúde sustentáveis.
Alta demanda emocional	Aumenta a vulnerabilidade ao desgaste psicológico, obstruindo a continuidade do atendimento e a adesão dos pacientes ao tratamento.
Rotatividade elevada de profissionais	Gera fragmentação do cuidado e instabilidade nas equipes; consequentemente, torna-se mais difícil a implementação de políticas públicas adequadas na assistência à saúde.
Contato frequente com a morte	Reduz a capacidade de tomar decisões clínicas e gera falhas na segurança do paciente, o que evidencia a necessidade de suporte psicológico adequado.
Falta de programas de suporte psicológico	Aumenta a incidência de transtornos mentais e afastamentos, reduzindo a qualidade da assistência e dificultando a implementação de políticas de saúde eficazes.
Dificuldade de comunicação entre equipes	Prejudica a execução de protocolos clínicos e compromete a segurança do paciente - problemas que impactam a efetividade das intervenções terapêuticas.
Mecanização do cuidado	Reduz a humanização da assistência: os profissionais afastam-se da relação terapêutica, e essa postura prejudica a adesão dos pacientes ao tratamento.
Sensação de fracasso profissional	Gera desmotivação e queda na produtividade dos profissionais, dificultando a implementação de políticas de saúde eficazes e sustentáveis.
Ausência de pausas e descanso adequado	Afeta a capacidade de concentração. Essa situação faz aumentar o risco de erros clínicos e compromete a segurança dos pacientes.
Impactos da pandemia de COVID-19	Intensifica a fadiga por compaixão devido ao aumento da carga de trabalho e ao luto coletivo, evidenciando falhas no suporte institucional aos profissionais de saúde.

* *ODS - Objetivos de Desenvolvimento Sustentável.*

Por fim, O Quadro 4 sintetiza os estudos selecionados de acordo com referência, população, método e desfecho do estudo.

**Quadro 4:** Síntese dos estudos incluídos na revisão de escopo

Ano/Referência	População	Método	Desfecho
Figley^(1)^	Profissionais que lidam com indivíduos em sofrimento	Livro	Conceitua e diferencia a fadiga por compaixão; apresenta sintomas e fatores de risco; e propõe estratégias de enfrentamento e prevenção para profissionais expostos ao sofrimento alheio.
Cocker F, Joss N (2016)^(2)^	Profissionais de saúde, emergência e serviços comunitários	Revisão sistemática	A fadiga por compaixão é comum nesses grupos, em razão da exposição constante ao sofrimento e à sobrecarga emocional, evidenciando a importância de suporte psicológico institucional e da redução da carga horária.
ONU (2015)^(3)^	População global	Documento oficial	Apresenta os 17 Objetivos de Desenvolvimento Sustentável (ODS) da Agenda 2030.
Duarte J, Pinto-Gouveia J (2017)^(4)^	Enfermeiros	Estudo transversal	Empatia excessiva e culpa estão fortemente associadas à fadiga por compaixão e ao *burnout*. A autorregulação emocional e a capacitação profissional surgem como fatores protetivos.
Garnett A, et al. (2023)^(9)^	Profissionais de saúde	Revisão de escopo	Mapeia estratégias de enfrentamento da fadiga por compaixão e aponta lacunas nas ações institucionais, reforçando a necessidade de diretrizes padronizadas.
Jilou V, et al. (2021)^(10)^	Profissionais da saúde	Revisão de escopo	Estratégias organizacionais, como treinamentos em inteligência emocional e suporte psicológico, atenuam a fadiga por compaixão, evidenciando a necessidade de ações institucionais para manter a qualidade assistencial.
Cardoso AA, Tomotani DYV, Mucci S (2023)^(11)^	Profissionais da saúde que lidam com pacientes no fim de vida	Estudo qualitativo	A exposição contínua ao sofrimento e à morte leva à exaustão emocional progressiva. Estratégias como suporte entre pares e supervisão emocional mostram-se essenciais à preservação da saúde mental dos profissionais.
Chen, et al. (2024)^(12)^	Profissionais da atenção primária	Estudo transversal	É essencial fortalecer o suporte institucional e recursos psicológicos para reduzir burnout e evitar perda de profissionais..
Phillips CS, Volker DL (2020)^(13)^	Enfermeiros oncológicos	Estudo qualitativo	A sobrecarga emocional e a exposição prolongada ao sofrimento geram desgaste mental significativo. O estudo ressalta a importância de práticas institucionais que equilibrem envolvimento emocional e distanciamento profissional saudável.
Mason VM, et al. (2014)^(14)^	Enfermeiros de trauma em UTI cirúrgica	Estudo-piloto	A fadiga por compaixão está vinculada ao sofrimento moral e ao aumento do engajamento laboral; programas de suporte psicológico podem fortalecer a resiliência emocional dos profissionais.
Zambrano SC, et al. (2014)^(15)^	Especialistas em medicina paliativa	Estudo qualitativo	A exposição contínua à morte afeta a saúde mental dos profissionais, resultando em altos níveis de exaustão e menor capacidade de empatia. A pesquisa recomenda melhorias nas supervisões psicológicas periódicas.
Pereira SM, et al. (2016)^(16)^	Profissionais de terapia intensiva	Estudo comparativo nacional	Trabalhar em terapia intensiva duplica as chances de esgotamento e fadiga por compaixão em comparação com os profissionais de cuidados paliativos. O estudo alerta para a necessidade de intervenções que reduzam a carga emocional desses profissionais.
Chang WP (2018)^(17)^	Enfermeiros clínicos	Estudo quantitativo	O suporte social adequado melhora a capacidade dos enfermeiros de lidar com a morte dos pacientes e reduz a fadiga por compaixão. Sua ausência está associada ao aumento do afastamento emocional dos profissionais.
Oliver A, et al. (2021)^(18)^	Profissionais de cuidados paliativos	Estudo transcultural	A prática do autocuidado é crucial para reduzir os efeitos da fadiga por compaixão e melhorar a qualidade de vida dos profissionais. O estudo sugere que as instituições de saúde promovam ações para favorecer o bem-estar dos trabalhadores.
Ko W, Kiser-Larson N (2016)^(19)^	Enfermeiros de oncologia	Estudo quantitativo	A exposição prolongada a casos graves de câncer leva a altos níveis de estresse ocupacional e fadiga. A pesquisa mostra que políticas institucionais de suporte psicológico são fundamentais para a atenção aos profissionais dessa área.
Laor-Maayany R, et al. (2020)^(20)^	Oncologistas	Estudo transversal	O luto mal elaborado e a sensação de fracasso ao lidar com pacientes em estágio terminal agravam a fadiga por compaixão. Estratégias como supervisão clínica e grupos de apoio podem minimizar esse impacto.
Pattison N, et al. (2020)^(21)^	Profissionais de saúde em cuidados críticos e paliativos	Estudo qualitativo	O *burnout* e a exaustão emocional afetam diretamente a capacidade dos profissionais de oferecer atendimento humanizado. O estudo destaca a necessidade de estratégias institucionais para preservar a saúde mental desses trabalhadores.
Moreira RS (2021)^(22)^	Profissionais da saúde no contexto da COVID-19	Estudo quantitativo	A pandemia aumentou os níveis de fadiga por sobrecarga de trabalho e mortes. O estudo aponta que protocolos de suporte psicológico podem reduzir danos emocionais em longo prazo.
Lago^(23)^	Profissionais de saúde no cuidado de pessoas em sofrimento físico e psíquico	Estudo qualitativo, revisão de literatura	Analisa o sofrimento psíquico dos profissionais de saúde no contexto do cuidado, aprofunda o conceito de fadiga por compaixão e propõe estratégias institucionais e subjetivas de enfrentamento.
Lago^(24)^	Profissionais de saúde em cuidados paliativos pediátricos	Revisão narrativa da literatura	Discute os desafios éticos, culturais, clínicos e organizacionais dos cuidados paliativos pediátricos no Brasil e no mundo.

## DISCUSSÃO

### A fadiga por compaixão como entrave à qualidade da assistência e ao ODS 3

A fadiga por compaixão, descrita como uma exaustão resultante da exposição contínua ao sofrimento alheio, tem sido mostrada como um fator determinante na degradação da saúde mental dos profissionais de assistência à saúde, afetando seu bem-estar individual, a qualidade dos serviços prestados e a segurança dos pacientes^(1)^. Ao longo da última década, investigações têm demonstrado que os impactos desse fenômeno extrapolam o domínio subjetivo, manifestando-se de forma concreta na elevação do absenteísmo, na rotatividade exacerbada e na deterioração das relações interpessoais dentro do ambiente organizacional^(2)^. Em um cenário de sobrecarga estrutural e escassez de suporte institucional, a fadiga por compaixão não só reduz a capacidade dos profissionais de responderem eficazmente às demandas dos pacientes, mas também aumenta o número de eventos adversos relacionados à assistência^(3,4,9)^.

A prevalência da fadiga por compaixão entre trabalhadores da saúde pode estar associada a múltiplos fatores organizacionais, incluindo jornadas extensas, volume excessivo de atendimentos e exposição recorrente a situações emocionalmente desafiadoras^(10)^. No contexto pandêmico, essa problemática tornou-se ainda mais alarmante, uma vez que o colapso dos sistemas de saúde e a intensificação das demandas exacerbaram os níveis de exaustão e desgaste psicológico dos profissionais^(11)^. Garnett et al.^(9)^ apontam que os profissionais atuantes na linha de frente de combate à COVID-19 apresentaram um aumento nos índices de fadiga por compaixão, secundário à exposição prolongada ao sofrimento humano sem suporte institucional adequado.

Além das repercussões individuais, as evidências demonstram que a fadiga por compaixão está diretamente relacionada ao aumento da propensão a erros médicos, ao declínio na qualidade do atendimento e à intensificação de sentimentos de despersonalização e cinismo entre os trabalhadores da saúde^(12,23)^. O ODS 3 destaca a importância de garantir a segurança do paciente e reduzir a mortalidade por causas evitáveis; entretanto, quando os profissionais estão submetidos a níveis elevados de exaustão emocional, reduz-se drasticamente a capacidade de prover um atendimento eficaz e humanizado^(13)^. A exaustão dos trabalhadores, portanto, não pode ser dissociada das metas estabelecidas para a saúde global, pois seu impacto sobre a qualidade assistencial diminui a efetividade das políticas públicas voltadas ao bem-estar populacional^(14)^.

A vulnerabilidade dos profissionais da saúde à fadiga por compaixão não se restringe ao ambiente hospitalar. De acordo com Cardoso, Tomotani e Mucci^(11)^, profissionais que atuam em cuidados paliativos e unidades de terapia intensiva apresentam índices elevados de sofrimento psíquico, intensificados pelo contato recorrente com a morte e pela necessidade de tomar decisões complexas sob intensa pressão. A ausência de estratégias institucionais voltadas à mitigação desse fenômeno contribui para um ciclo de desgaste progressivo, no qual a exaustão emocional torna-se um fator determinante para o afastamento do trabalho e saída precoce da profissão^(15)^. Essa perda de profissionais qualificados prejudica a continuidade do atendimento e agrava a desigualdade no acesso à saúde, dificultando a implementação das metas do ODS 3 e fragilizando a sustentabilidade dos sistemas assistenciais.

A relação entre a fadiga por compaixão e a efetivação do Objetivo de Desenvolvimento Sustentável 3 (ODS 3) torna-se evidente ao se observar que a estabilidade das equipes de saúde é um dos pilares para garantir um atendimento equitativo e de qualidade^(16,17)^. A Agenda 2030, proposta pela Organização das Nações Unidas (ONU), estabelece que o acesso à saúde não deve se limitar à ampliação de serviços, mas incluir a oferta de cuidados seguros, humanizados e sustentáveis^(3)^. Porém, a fadiga por compaixão é uma barreira para o alcance dessa meta, pois profissionais exaustos tendem a apresentar redução da empatia, dificuldades na comunicação clínica e maior predisposição a erros assistenciais; esses problemas agravam-se em contextos de alta demanda, como emergências hospitalares e unidades de terapia intensiva, onde a sobrecarga emocional atinge níveis extremos e há menos oportunidades para recuperação psicológica^(10,11,18,24)^.

Os impactos da fadiga por compaixão sobre a segurança do paciente são amplamente documentados. Ko e Kiser-Larson^(19)^ identificaram que instituições com elevados índices desse fenômeno entre os profissionais enfrentam taxas crescentes de absenteísmo, rotatividade e fragmentação da assistência, de modo que a continuidade do cuidado é afetada negativamente. A rotatividade de equipe, por sua vez, influencia diretamente a adesão dos pacientes ao tratamento, pois a falta de vínculo terapêutico reduz a confiança no sistema de saúde e dificulta a implementação de estratégias terapêuticas de longo prazo^(20)^.

Estudos conduzidos entre 2020 e 2021 apontaram que profissionais de saúde com altos níveis de fadiga por compaixão também relataram menor satisfação no trabalho e um comprometimento profissional reduzido. Além disso, verificou-se um aumento na intenção de deixar a organização, evidenciado pela maior propensão à rotatividade. Em outro contexto, observou-se que a fadiga por compaixão impactou negativamente as intenções comportamentais de enfermeiros no atendimento a pacientes com COVID-19, o que pode minar a qualidade do cuidado prestado^(9)^.

Percebe-se que, quando os trabalhadores da saúde não encontram suporte adequado, há um aumento no risco de *burnout* e tendência à mecanização dos atendimentos, afastando a prática assistencial dos princípios estabelecidos pelo ODS 3, que preconiza uma assistência centrada no paciente e focada no bem-estar populacional^(12)^.

A sobrecarga emocional provoca queda na qualidade do atendimento, assim como na segurança dos processos clínicos, gerando um ambiente mais propenso a falhas operacionais. Mason et al.^(14)^ apontam que profissionais submetidos a longos períodos de exaustão emocional demonstram redução na capacidade de tomar decisão, dificuldades no julgamento clínico e maior vulnerabilidade a equívocos na administração de medicamentos, interpretação de exames e manejo de dispositivos médicos. A sobrecarga emocional enfraquece a cognição dos profissionais, tornando-os menos atentos a pequenos detalhes que poderiam indicar a deterioração do quadro do paciente^(16)^.

De acordo com Garnett et al.^(9)^, os efeitos negativos da pandemia de COVID-19 sobre a saúde mental e bem-estar dos profissionais de saúde continuam a limitar suas atividades e funções, contribuindo para o desenvolvimento de fadiga por compaixão. A escassez de equipamentos de proteção individual agravou a disseminação do vírus e intensificou o medo entre esses profissionais de contrair e transmitir a doença para colegas, pacientes e familiares. Esse cenário resultou em aumento de ausências, licenças médicas prolongadas e maior rotatividade de pessoal. A sobrecarga intensificou-se diante da escassez de equipes, das constantes alterações nos fluxos de trabalho e da prestação contínua de cuidados a pacientes gravemente enfermos, frequentemente com desfechos fatais.

Dessa forma, a negligência das instituições em relação ao bem-estar dos trabalhadores não apenas afeta a saúde ocupacional, mas também gera prejuízo na efetividade das intervenções terapêuticas e na segurança dos pacientes.

Implementar estratégias institucionais para reduzir a fadiga por compaixão representa um passo fundamental para a efetivação do ODS 3. Garnett et al.^(9)^ destacam que políticas voltadas à redução da carga de trabalho, ampliação do suporte psicológico e capacitação dos profissionais em estratégias de enfrentamento emocional podem resultar em melhorias significativas na retenção de trabalhadores e na qualidade assistencial. Cardoso, Tomotani e Mucci^(11)^ reforçam que a fadiga por compaixão está diretamente associada ao aumento da mortalidade por causas evitáveis, pois profissionais desgastados demonstram menor capacidade de resposta a emergências e menor assertividade na conduta clínica. Portanto, assegurar que os trabalhadores da saúde atuem em condições adequadas não é uma mera questão ocupacional, mas uma estratégia essencial para a redução da morbimortalidade e concretização dos princípios estabelecidos pelo ODS 3^(23)^.

Além disso, a fadiga por compaixão afeta a equidade no acesso à saúde, pois inviabiliza a capacidade dos sistemas assistenciais de manter equipes estáveis e suficientemente capacitadas para atender à crescente demanda populacional. Chen et al.^(12)^ analisaram como a exaustão emocional impacta profissionais da atenção primária: em locais onde a sobrecarga de trabalho é intensa e o suporte institucional é insuficiente, a rotatividade de equipe resulta em descontinuidade dos atendimentos e desigualdade no acesso a serviços essenciais. Essa situação é particularmente preocupante em países de baixa e média renda, onde a carência de profissionais de saúde já representa um desafio à universalização do atendimento^(17)^.

A implementação de políticas públicas e organizacionais voltadas à prevenção da fadiga por compaixão deve ser encarada como um dos eixos centrais para a concretização do ODS 3. Figley^(1)^ defende que a resiliência emocional dos profissionais não deve ser tratada como uma responsabilidade individual, mas como uma construção coletiva que envolve mudanças institucionais e o fortalecimento de práticas direcionadas à proteção da saúde mental no ambiente de trabalho. A inclusão de programas de suporte psicológico, treinamento para gerenciamento do estresse ocupacional e medidas que promovam jornadas laborais mais equilibradas são fundamentais para garantir que os trabalhadores estejam física e emocionalmente aptos a exercerem sua função de maneira ética, segura e eficiente^(21)^.

A transformação desse cenário demanda um compromisso de gestores, formuladores de políticas públicas e instituições de ensino em saúde, com a promoção de um modelo assistencial no qual se assegure que os princípios do ODS 3 sejam aplicados não apenas à população atendida, mas também aos profissionais responsáveis por garantir essa assistência. Mais do que ampliação do acesso, a sustentabilidade dos sistemas de saúde depende da implementação de estratégias voltadas à construção de um ambiente de trabalho que proteja o profissional da exaustão e permita a continuidade de um atendimento seguro, humanizado e alinhado às necessidades da sociedade^(21,22)^.

Portanto, enfrentar a fadiga por compaixão vai além da necessidade dos profissionais de saúde: é uma medida essencial para a sustentabilidade dos sistemas assistenciais e para a construção de um modelo de atenção em conformidade com os princípios do ODS 3. Apenas estratégias estruturadas e sustentáveis poderão garantir que o compromisso global de assegurar bem-estar e saúde a todos não permaneça apenas como um ideal, mas se concretize como uma realidade acessível e equitativa para todas as populações.

### Limitações do estudo

As limitações deste estudo envolvem, inicialmente, o fato de que nem todos os fatores relacionados à fadiga por compaixão e sua interface com o Objetivo de Desenvolvimento Sustentável 3 (ODS 3) foram abordados de forma específica e aprofundada na literatura analisada. Observou-se também uma escassez de ensaios clínicos e revisões sistemáticas diretamente voltados à temática, o que dificultou a identificação de evidências sólidas e conclusivas sobre estratégias eficazes para mitigar a fadiga por compaixão. Essa limitação foi acentuada pela restrição de acesso ao texto completo de alguns artigos identificados nas bases de dados consultadas. Ademais, a heterogeneidade dos dados entre os estudos incluídos pode ter impactado a consistência e a aplicabilidade dos achados, especialmente diante das particularidades dos diferentes sistemas de saúde contemplados.

### Contribuições para a área

Este estudo oferece uma visão detalhada sobre os fatores associados à fadiga por compaixão, ampliando a compreensão de como esse fenômeno impacta diretamente a implementação do ODS 3, especialmente no que diz respeito à qualidade do atendimento e à segurança dos pacientes. Ele contribui ao identificar lacunas no conhecimento, que podem direcionar novas pesquisas sobre o tema; e ao destacar a necessidade de estratégias estruturais no ambiente de trabalho para apoiar os profissionais de saúde. Além disso, este trabalho reforça a importância de incorporar a fadiga por compaixão nas políticas de saúde pública, com foco na melhoria das condições de trabalho e na promoção da saúde mental dos trabalhadores, a fim de garantir a efetividade das metas globais de saúde e bem-estar.

## CONSIDERAÇÕES FINAIS

Este estudo evidenciou que a fadiga por compaixão representa um desafio significativo para a sustentabilidade e qualidade dos sistemas de saúde, impactando diretamente tanto os profissionais da área quanto os pacientes e a efetivação do Objetivo de Desenvolvimento Sustentável 3 (ODS 3). O desgaste emocional e psicológico dos trabalhadores de saúde compromete a segurança do paciente, aumenta a incidência de erros clínicos e contribui para a fragmentação da assistência, demonstrando que essa problemática ultrapassa a esfera individual e reflete falhas estruturais nos ambientes de trabalho.

Os achados revelam que a fadiga por compaixão está associada a fatores como jornadas extenuantes, escassez de suporte institucional e exposição prolongada ao sofrimento alheio, o que pode levar ao aumento da rotatividade dos profissionais, ao absenteísmo e à redução da qualidade assistencial. Em um contexto de sobrecarga dos sistemas de saúde, como observado na pandemia de COVID-19, essas questões tornam-se ainda mais alarmantes.

A interseção entre a fadiga por compaixão e o ODS 3 destaca a necessidade de estratégias estruturais para suavizar seus impactos. A efetivação das metas globais de saúde depende não apenas de ampliar o acesso à assistência, mas também de implementar condições adequadas para que os profissionais desempenhem suas funções de maneira segura e eficaz. A falta de iniciativas voltadas para a proteção da saúde mental dos trabalhadores pode prejudicar a sustentabilidade dos serviços de saúde e a qualidade da assistência prestada.

Diante disso, este estudo reforça a importância de políticas institucionais voltadas à prevenção da fadiga por compaixão. Medidas como redução da carga horária, apoio psicológico, capacitação em estratégias de enfrentamento emocional e promoção de um ambiente de trabalho mais humanizado são essenciais para minimizar os impactos desse fenômeno. A adoção dessas ações pode melhorar a qualidade assistencial, reduzir a vulnerabilidade dos profissionais e promover um sistema de saúde mais resiliente e equitativo.

Conclui-se que enfrentar a fadiga por compaixão não é apenas uma questão de bem-estar ocupacional, mas também um requisito para a efetividade das políticas de saúde pública. Somente o compromisso coletivo de gestores, formuladores de políticas e profissionais da saúde poderá garantir que o ODS 3, fundamentado no compromisso com o acesso universal à saúde e promoção do bem-estar para todos, seja mais do que um ideal global, mas uma realidade acessível a todas as populações.

## Data Availability

Os dados de pesquisa estão disponíveis no corpo do artigo.
