# Supplementary material for: Erratum: Compassion fatigue: a challenge to comprehensive health care and SDG 3 under the 2030 Agenda
Source: Rev Bras Enferm. 2026 Aug 7;79:e2026e0320250175. doi: 10.1590/0034-7167.202679e0320250175 (PMC13450936; doi:10.1590/0034-7167.202679e0320250175)
Supplement: Related texts

# Correction: Errata: Fadiga por compaixão: desafio à saúde integral e ao ODS 3 da Agenda 2030

**DOI:** 10.1590/0034-7167.202679e0320250175pt

No artigo “Fadiga por compaixão: desafio à saúde integral e ao ODS 3 da Agenda 2030”, com número DOI: https://doi.org/10.1590/0034-7167-2025-0175pt, publicado no periódico Revista Brasileira de Enfermagem, 2026;79:e20250175, na página 9:

Onde se lia:

**FOMENTO**

Fundação de Amparo à Pesquisa do Estado de Minas Gerais (FAPEMIG).

Lê-se:

**FOMENTO**

Fundação de Amparo à Pesquisa do Estado de Minas Gerais (FAPEMIG). Processo APQ-04716-24, aprovado pelo Edital Nº 009/2024 - Fortalecimento e consolidação da pesquisa na UEMG E UNIMONTES.

Profa. Dra. Dulce Barbosa

*Editora-Chefe*
